# Selective Recovery of Critical Minerals from Simulated Electronic Wastes Via Reaction‐Diffusion Coupling

**DOI:** 10.1002/cssc.202402372

**Published:** 2025-02-18

**Authors:** Qingpu Wang, Yucheng Fu, Erin A. Miller, Duo Song, Philip J. Brahana, Andrew Ritchhart, Zhijie Xu, Grant E. Johnson, Bhuvnesh Bharti, Maria L. Sushko, Elias Nakouzi

**Affiliations:** ^1^ Physical and Computational Sciences Directorate Pacific Northwest National Laboratory Washington 98109 Seattle United States; ^2^ Physical and Computational Sciences Directorate Pacific Northwest National Laboratory Washington 99354 Richland United States; ^3^ National Security Directorate Pacific Northwest National Laboratory Washington 99352 Richland United States; ^4^ Cain Department of Chemical Engineering Louisiana State University Louisiana 70803 Baton Rouge United States

**Keywords:** Critical elements, reaction-diffusion, crystallization, separations, interfaces

## Abstract

Atom‐ and energy‐efficient chemical separations are urgently needed to meet the surging demand for critical materials that has strained supply chains and threatened environmental damage. In this study, we used reaction‐diffusion coupling to separate iron, neodymium, and dysprosium ions from model feedstocks of permanent magnets, which are typically found in electronic wastes. Feedstock solutions were placed in contact with a hydrogel loaded with potassium hydroxide and/or dibutyl phosphate, resulting in complex precipitation patterns as the various metal ions diffused into the reaction medium. Specifically, we observed the precipitation of up to 40 mM of iron from the feedstock, followed by the enrichment of 73 % dysprosium, and the extraction of >95 % neodymium product at a further distance from the solution‐gel interface. We designed a series of experiments and simulations to determine the relevant ion diffusivities, *D_Nd_* =5.4×10^−10^ and *D_Dy_* =5.1×10^−10^ m^2^/s, and precipitation rates, *k_Nd_* =1.0×10^−5^ and *k_Dy_* =5.0×10^−3^ m^9^ mol^−3^ s^−1^, which enabled a numerical model to be established for predicting the distribution of products in the reaction medium. Our proof‐of‐concept study validates reaction‐diffusion coupling as an effective and versatile approach for critical materials separations, without relying on ligands, membranes, resins, or other specialty chemicals.

## Graphical abstract

We achieve the facile separation of Fe, Dy, and Nd from model solutions of electronic waste using reaction‐diffusion coupling. Using a suite of X‐ray imaging, characterization, atomistic, and continuum simulations, we develop a predictive model for the distribution of the components in the reaction medium.

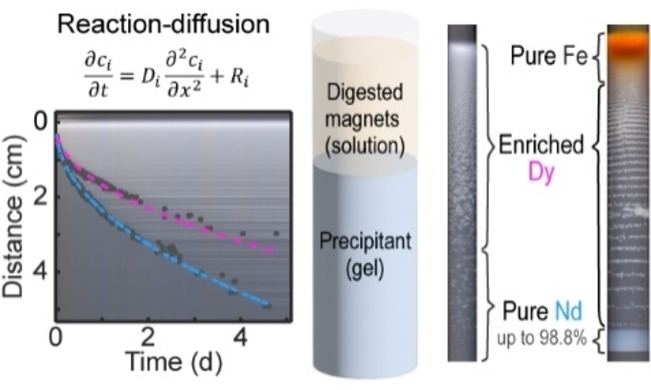

## Introduction

The challenging reality is that transitioning to a green economy will be powered by critical materials, whose sourcing is fraught with environmental, ethical, and supply chain concerns.[^1^, ^2^] These critical materials, such as neodymium (Nd) and dysprosium (Dy), are key components in wind turbines, electric vehicle motors, and hard disk drives. Meeting the surge in demand will require a fundamental understanding of chemical separations that yields a new generation of atom‐ and energy‐efficient separation technologies. Traditionally, rare earth elements (REEs) are mined from mineral ores through complex energy‐ and chemical‐intensive processes.^[3]^ Upon extraction, the ores are concentrated through flotation methods,[^4^, ^5^] roasted to decompose the carbonate minerals, leached in acid, re‐precipitated in alkaline solutions to extract impurities such as iron, and processed using solvent extraction to recover the target REEs.^[6]^ While this separation process is widely deployed at the industrial scale, it has multiple disadvantages, including large chemical requirements, the generation of hazardous byproducts, the use of toxic organic extractants, and low efficiency for separating dilute feedstocks.^[3]^

Accordingly, there is increasing interest in sourcing REEs from widely available unconventional feedstocks that are more environmentally sustainable and address mounting supply chain challenges.^[3]^ One prime candidate is electronic waste, of which the world produces >50 million tons annually, valued at >$60 billion. Only 20 % of these waste materials are currently recycled.^[3]^ Specifically, neodymium‐iron‐boron (NdFeB) permanent magnets represent a substantial quantity of electronic waste due to their rapidly increasing use in electric motors. NdFeB magnets are composed of 25–35 % REEs (mainly Nd, Pr, and Dy), 60–70 % iron (Fe), and∼1 % boron (B),^[7]^ whose separation requires substantial energy consumption.^[8]^

In a recent National Academies report, the Committee on a Research Agenda for a New Era in Separation Science stated the need for “breakthroughs in the next generation of separation science.”^[9]^ Promising technologies include separations using proteins,^[10]^ electrorefining,^[11]^ ligand‐assisted^[12]^ and counter‐current chromatography,^[13]^ as well as machine learning optimization of separation materials.^[14]^ However, most of these approaches involve specialty chemicals that are often difficult or costly to scale up or adapt to complex feedstock compositions. For example, a significant challenge in electronic waste recycling is the high concentration of residual iron (Fe), which compromises the performance of the separation membrane or device.^[15]^

Recently, we demonstrated proof of concepts for separating critical materials using simple precipitation reactions without relying on organic solvents, resins, membranes, or other specialty chemicals.[^16^, ^17^, ^18^] Our approach was inspired by the classic Liesegang experiment, where the non‐linear coupling of reaction and diffusion produces unexpected precipitation patterns.^[18]^ Specifically, the feedstock solution is placed in contact with a hydrogel that is loaded with a precipitating agent. As the various metal ions diffuse into the gel, the fastest‐reacting species locally consume the precipitating agent, producing a pure product. The competing products then precipitate sequentially according to the interplay of reaction kinetics and ion diffusion. Using this approach, we reported the enrichment of manganese from recycled battery cathodes using sodium hydroxide.^[18]^ Separation of precipitates was also demonstrated for copper and cobalt oxalates in a flow‐driven system.^[20]^

In this study, we report the selective reaction‐diffusion separation of critical materials from model feedstock solutions of recycled magnets. First, we demonstrate the separation of Nd and Dy, developing a model that predicts the distribution of these components in the reaction medium. Secondly, we show that reaction‐diffusion separations may be adaptable to more complex feedstocks, successfully pre‐screening substantial amounts of residual iron before separating components of the REE mixture. Our findings demonstrate that reaction‐diffusion coupling is a highly versatile approach for the enrichment of critical minerals from widely available unconventional feedstocks.

## Results and Discussion

### Sequential Precipitation of Neodymium and Dysprosium

**Figure 1:**
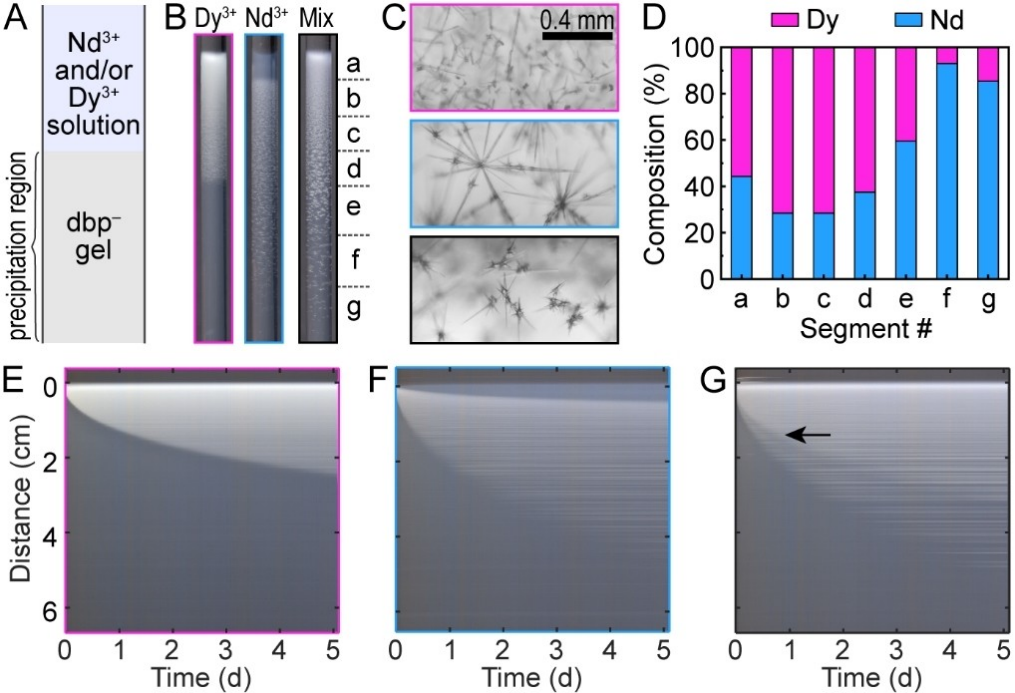
Separation of Nd and Dy via reaction‐diffusion coupling. (A) Schematics of the experimental setup. (B) Photographs of the resulting precipitate patterns for individual Dy^3+^, Nd^3+^, and mixed salts, with border color coded with magenta, blue, and black, respectively. (C) Representative optical micrographs of precipitate crystals in the Dy‐only (magenta), Nd‐only (blue), and mixed‐salt (black) experiments. (D) Molar composition along the precipitate from a mixed‐salt experiment measured by ICP‐MS. (E−G) Time‐space plots of Dy‐only (E), Nd‐only (F), and mixed‐salt (G) experiments.

In the first series of experiments, we investigated model solutions with a Nd : Dy molar ratio of 4 : 1, which is typical for neodymium‐based permanent magnets.[^21^, ^22^] Specifically, we used individual and mixed salt solutions of 40 mM neodymium(III) chloride and 10 mM dysprosium(III) chloride. These solutions were placed in a tube on top of a layer of agarose gel loaded with 10 mM potassium dibutyl phosphate (Kdbp), a precipitating agent with a promising separation efficiency for these components.^[23]^ Within a few seconds of adding the solution, a white precipitate began to form near the solution‐gel interface as ions diffused across the boundary between the two media. Because of the higher reactant concentration in the solution compared to the gel, the precipitate primarily formed in the gel, driven by the concentration gradient (Figure 1A).

The pattern and dynamics of the precipitate formation varied with the solution composition (Figure 1B). For example, the DyCl_3_‐only solution produced a continuous precipitate layer with a decreasing density that extended 26 mm into the gel within 5 days. For the NdCl_3_‐only solution, the precipitate initially formed near the solution‐gel interface, but continuously dissolved and re‐precipitated (Movie S1 in the Supporting Information, SI), creating a propagating front that left in its wake a uniform translucent layer. At *t*=5 days, this layer had a length of 4.2 mm, preceded by a longer precipitate layer of approximately 41 mm. The distribution of solid material in the precipitate was relatively sparse, with decreasing density at further distances from the solution‐gel interface. By comparison, the mixed salt solution resulted in a longer precipitate layer, extending 51 mm into the gel. We observed only slight dissolution near the interface, but the pattern was dominated by the growth of new crystals (Figures 1B and S1).

Optical micrographs of the various crystal morphologies in the three solution conditions are presented in Figure 1C. The Dy‐only solution produced funnel‐ and needle‐shaped crystals that were individually dispersed and relatively densely packed. The Nd‐only solution produced longer needles that emerged from a seed crystal and were more sparsely distributed. Aggregates of relatively short, pointy needles were observed in the mixed‐salt experiment (Figure 1C). For all these conditions, the average particle size increased with increasing distance from the solution‐gel interface (Figure S1). This observation is consistent with previous studies on precipitation in reaction‐diffusion systems[^24^, ^25^] and may be ascribed to the change in supersaturation during the process.^[26]^ Initially, the high flux of ions into the gel results in a high nucleation rate that produces a large number of relatively small particles. As the ions are consumed, the nucleation rate decreases significantly. At this stage, precipitation is dominated by the growth of existing particles rather than the nucleation of new particles, resulting in the formation of fewer, larger crystals.

The observed spatial gradients in particle morphology, size, and density suggest the possibility of compositional changes across the precipitate layer. To evaluate this prospect, we quantified the elemental composition of the precipitates using inductively coupled plasma mass spectrometry (ICP‐MS)ϵ measurements. Gel segments of approximately 7 mm in length were extracted from the mixed‐salt experiment (Figure 1C). The selection of the exact segment locations was informed by qualitative features in the individual salt experiments (Figure S2). We observed a Dy‐rich region – with up to 71.6 % Dy – in segments a–d. This region extended 26 mm into the gel which was comparable to the total precipitation length of the Dy‐only experiment (Figure 1D). Beyond this Dy‐rich layer, the Nd ratio increased significantly, reaching 93.1 % in segment f. These results furnish a low‐energy pathway for Dy enrichment and subsequent Nd separation from a mixed feedstock solution via reaction‐diffusion coupling.

To examine the dynamics of precipitate formation, we constructed time‐space plots by collecting consecutive images of the experiment, averaging individual images across the tube radius, and stacking the resulting 1D profiles (Figures 1E–G and Movie S2). In these time‐space plots, the precipitates are visualized as a white propagating front against the gray background of the reaction medium (Figure S4). The plot for the Dy‐only experiment (Figure 1E) showed a single front corresponding to the propagation of the precipitate in the gel medium. The Nd‐only plot showed a leading precipitation front and an inner front ascribed to the slow dissolution of the as‐formed precipitate (Figure 1F). This inner front left a residue of partially dissolved crystals observed in optical micrographs (Figure S3). The particles remained stationary once formed and were visualized in the time‐space plots as horizontal lines with slight gaps in between, particularly for the sparser Nd precipitates.

For the mixed‐salt experiment, the time‐space plot also showed two propagating fronts. However, both fronts were ascribed to precipitation – rather than dissolution – for two key reasons: Firstly, the precipitates were denser within the inner front (black arrow, Figure 1G) indicating the formation of additional solid material. Secondly, the inner and outer fronts were approximately overlapping with the precipitation fronts in the Dy‐only and Nd‐only experiments, respectively (Figure S4). By tracking the precipitation fronts, we were able to evaluate the propagation rates of the precipitation fronts. We found that the experimental data agreed reasonably well with square root fits (Figure S4).

### Predictive Simulations of Neodymium and Dysprosium Distributions

Following the preliminary characterization of the precipitation dynamics, we pursued rigorous simulations to predict the distribution of Nd and Dy within the final products. The model was informed by multiple experimental steps, which enabled the extraction of the key numerical parameters, namely the diffusion coefficients for the two metal ions (*D_Nd_* and *D_Dy_*) and the precipitation reaction rates of the corresponding dibutyl phosphates (*k_Nd_* and *k_Dy_*). The numerical simulations were complemented with molecular simulations to reveal the role of solvation and ion‐ion correlation on the effective diffusivity of Nd and Dy.

**Figure 2:**
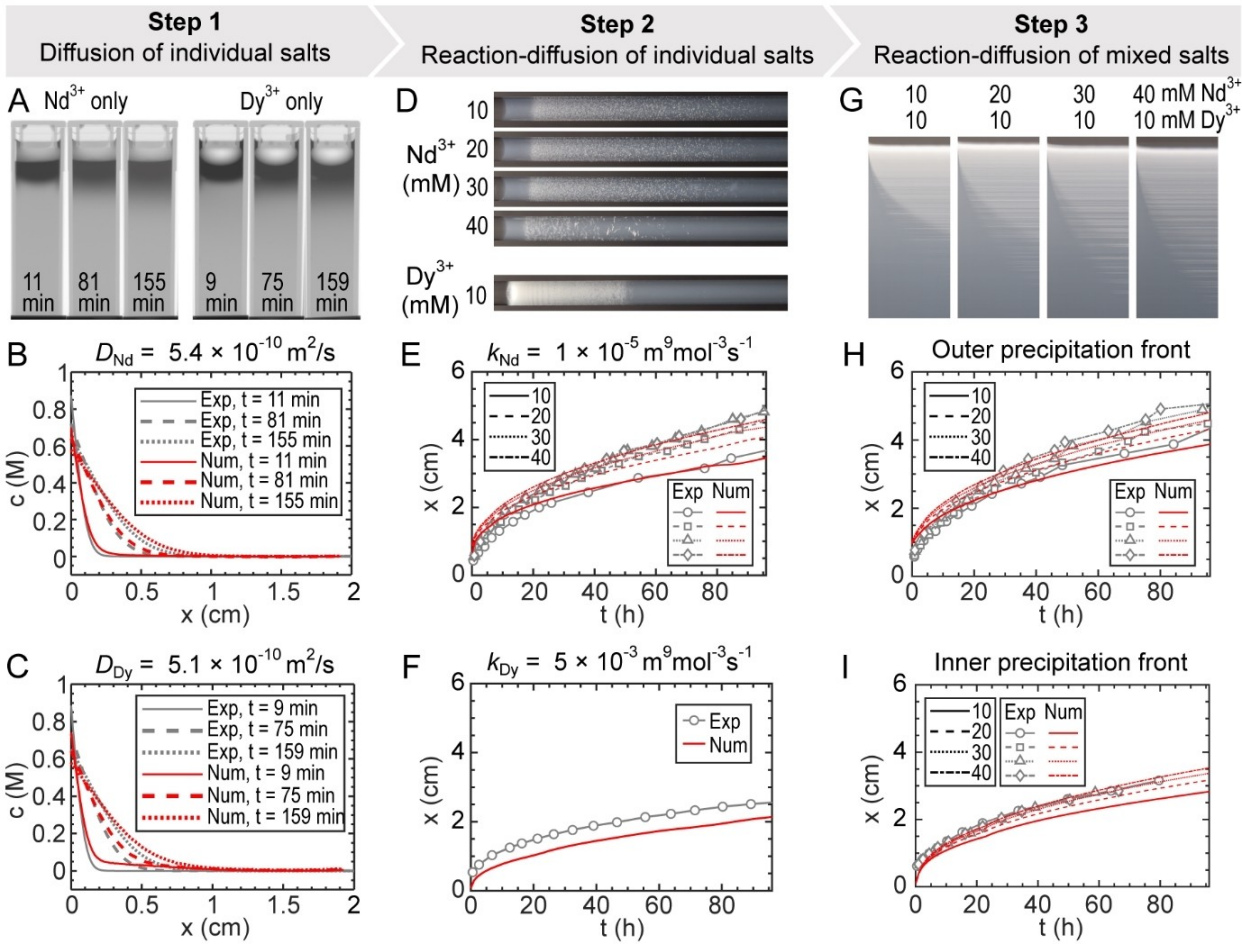
Building a numerical model for predicting the distribution of Nd and Dy in the reaction medium. (A) X‐ray images of NdCl_3_ and DyCl_3_ diffusion into agarose gel, with no precipitation reactions. (B, C) Evolution of concentration profiles from experimental (black) and best‐fit simulation (red) data for Nd (B) and Dy (C). (D) Images of reaction‐diffusion experiments from NdCl_3_ and DyCl_3_ single‐salt solutions diffusing into gel loaded with Kdbp. (E, F) Precipitation fronts acquired from experimental (gray) and best‐fit simulation (red) data for Nd (E) and Dy (F). (G) Time‐space plots showing precipitation fronts from mixed salt experiments. (H) Outer precipitation front and (I) inner precipitation front acquired from experimental (gray) and directly calculated simulation (red) data. The numbers in the legends of E,H, and I represent the concentration of Nd^3+^.

In the first step, we attempted to measure the diffusivity of Nd^3+^ and Dy^3+^ ions in an agarose gel medium that was free from the precipitating agent. We monitored the evolution of ion concentration profiles using X‐ray imaging (Figures 2A and Figure S5). Note that this technique is routinely used in airport security checks and particularly suited for imaging heavy metals that are otherwise optically transparent and thus been used to determine ion diffusivity in gel media.[^27^, ^28^, ^29^, ^30^] To facilitate the X‐ray image acquisition, we used relatively high salt concentrations of 1 M placed in rectangular cuvettes rather than cylindrical tubes. Concentration profiles at multiple time points reveal ions penetrating approximately 1 cm into the gel layer over a period of ~160 mins (Figures 2B, C). Calibration curves were collected to convert the intensity data to ion concentrations (Figure S19). Notice that the bulk concentrations in the solutions were depleted by 28.2 % and 35.3 % for Nd^3+^ and Dy^3+^, respectively, during the experiment. Due to the complexities of source depletion and reactor geometry, it was not possible to use an analytical expression to accurately determine the ion diffusivities.

We thus performed numerical simulations based on the diffusion equation (Fick′s law) by replicating the experimental parameters (see SI for details). The simulations were benchmarked against the experimental concentration profiles to recover diffusion coefficients of 5.4×10^−10^ m^2^/s for Nd^3+^ and 5.1×10^−10^ m^2^/s for Dy^3+^ (Figures 2B,C and S6). These values were slightly lower than the reported diffusivities in bulk aqueous media of 6.2×10^−10^ and 5.8×10^−10^ m^2^/s for Nd^3+^ and Dy^3+^, respectively.^[31]^

The effective diffusivities were then used to calculate the gel porosity ϵ as described by the Millington and Quirk model,^[32]^ *ϵ^4/3^=D/D_0_*. We obtained *ϵ*=0.90 which is comparable to literature reports for hydrogels such as agarose^[33]^ and silica.^[34]^ As such, we anticipate that confinement in the gel pores did not drastically alter the ion diffusivities. Instead, ion diffusion was slowed down by 12–13 %. This behavior was starkly different from our visual observations of the precipitate particles, which were completely stationary in the gel. The rationale is that the gel pores with sizes ranging between tens to hundreds of nanometers[^35^, ^36^] are sufficiently large for free and almost unhindered ion diffusion, but too small for solid particles to move freely without colliding with the gel fibers.^[37]^

These hypotheses were corroborated using calculations of the effective diffusivities of Nd^3+^ and Dy^3+^ in pure solutions and their mixtures under confinement. Simulations of ion diffusivity in a 15 nm pore revealed that ion solvation is the main contribution to the potential of mean force (PMF) of Nd^3+^ and Dy^3+^ ions in dilute solutions (10 mM). Solvation interactions constitute 53.7 % and 50.3 % of the PMF of Nd^3+^ and Dy^3+^, respectively. The relative contribution of ion correlation forces increases with concentration and becomes 13.7 % and 14.6 % of cation PMF in 1 M solutions. The increase in ion‐ion correlation leads to a decrease in the effective diffusivity of the cations in pure solutions (Table 1). Partial disruption of ion‐ion correlation in 0.5 M NdCl_3_–0.5 M DyCl_3_ mixed solutions manifests in the increase in diffusivities of each cation compared to their pure solutions. In contrast, the Nd^3+^ and Dy^3+^ diffusivities decrease in a dilute mixture relative to pure solutions due to a 1.9 % and 2.5 % reduction in solvation PMF, respectively.

**Table 1:** Effective diffusivities in NdCl_3_ and DyCl_3_ electrolytes and their mixtures.

Systems	Diffusivity (×10^−10^ m^2^/s)	
	Nd^3+^	Dy^3+^
10 mM NdCl_3_	5.58	–
1 M NdCl_3_	5.33	–
10 mM DyCl_3_	–	5.26
1 M DyCl_3_	–	5.03
5 mM NdCl_3_+5 mM DyCl_3_	5.47	5.13
0.5 M NdCl_3_+0.5 M DyCl_3_	5.37	5.05

After characterizing ion diffusion, we attempted to measure the reaction rates of the Nd and Dy precipitation. Here we used reaction‐diffusion experiments of the individual salt solutions of NdCl_3_ (10–40 mM) and DyCl_3_ (10 mM) placed on top of the agarose gel loaded with 10 mM Kdbp. The precipitation fronts were straightforward to extract from optical imaging, as demonstrated in Figures 1E–G. In principle, this precipitation front proceeds at a rate determined by the coupling of ion diffusion and precipitation kinetics. Accordingly, the spatial and temporal evolution of reactant and precipitate species were evaluated using the reaction‐diffusion equation (Eq. S2).

Since the ion diffusivities were already acquired from the previous step, the only remaining fitting parameter was the precipitation rate coefficient for the corresponding Nd and Dy precipitates. We found optimal agreement between the simulations and experimental data using *k_Nd_* =1.0×10^−5^ m^9^ mol^−3^ s^−1^ and *k_Dy_* = 5.0×10^−3^ m^9^ mol^−3^ s^−1^. (Figures S7,S8 and modeling details in the SI) Importantly, Dy precipitation was ~500 times faster than Nd; a vast difference in the reaction rates compared to the diffusivities, which only differed by 5.8 %.

The third step was to validate the numerical model for the case of mixed feedstock solutions. We simulated the precipitation fronts from mixed solutions of NdCl_3_ (10–40 mM) and DyCl_3_ (10 mM) diffusing into a gel medium loaded with Kdbp (10 mM), mirroring the experimental conditions for data reported in Figure 1 and Figures 2G (also see Figure S9). Similar to the experiments, we observed two precipitation fronts in the simulation, an outer front dominated by the Nd precipitate (Figure 2H) and a slower inner front that consisted mostly of Dy precipitate (Figure 2I). Notice that no fitting was performed in this step – these results were direct calculations using numerical parameters recovered in the two previous steps from the individual salt solutions. Accordingly, the simulation results agreed reasonably well with the experimental data, indicating that the reaction‐diffusion model presents a powerful tool for predicting the distribution of the various components and optimizing the conditions for chemical separations. The limitations of the model are described in the Discussion section below.

### Precipitation with Gel Containing Two Reactants

We tested the robustness and versatility of reaction‐diffusion separations by increasing the complexity of the feedstock solution. Specifically, we investigated the effect of the presence of iron(III) chloride (FeCl_3_) in the feedstock, since dissolved magnets typically contain residual amounts of iron that compromise the separation efficiency and purity. Our approach used two precipitating reagents in the gel to interact primarily with the iron and REEs. We selected KOH as the additional precipitating agent because it forms hydroxide precipitates with non‐alkali metal ions and is a widely used commodity chemical in industrial separations.

**Figure 3:**
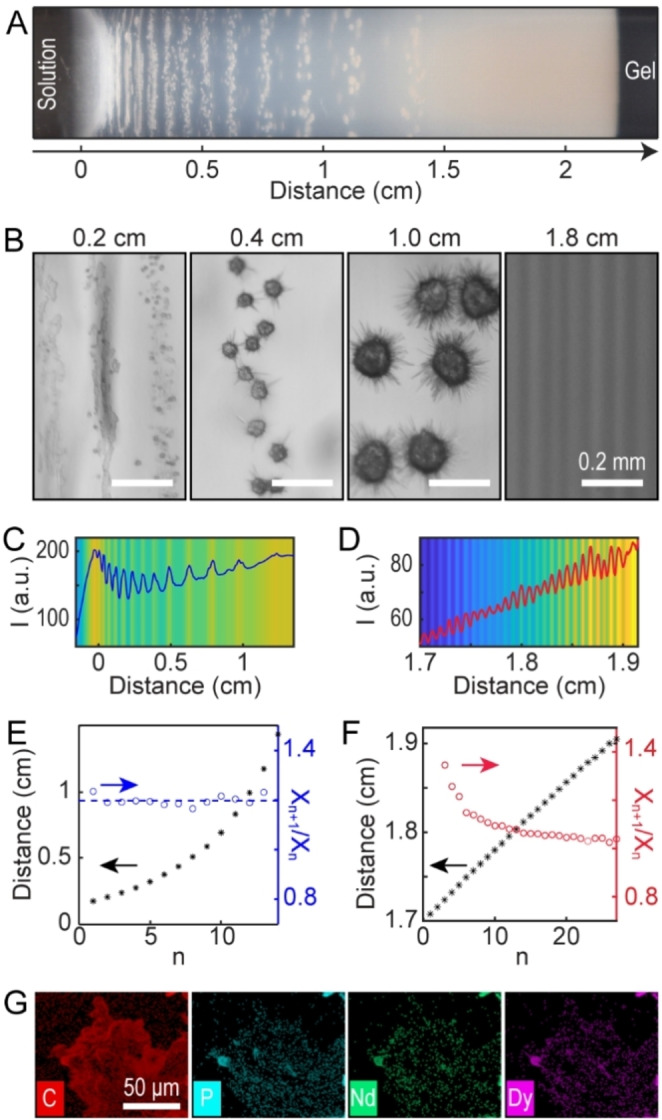
Reaction‐diffusion coupling with two precipitating agents in the gel. (A) Photograph of the precipitate pattern with mixed Nd^3+^ and Dy^3+^ in the aqueous solution and a mixture of dbp^−^ and OH^−^ in the gel. (B) Optical micrographs of precipitates at distances from the solution‐gel interface as indicated on top of the panels. All scale bars: 0.2 mm. (C−F) Intensity of averaged image cropped at different regions and spacing analysis for the large‐scale (C,E) and small‐scale (D,F) precipitate bands. The hollow circles mark the ratio of two consecutive bands and refer to the right‐side Y‐axes in E and F. (G) EDS maps of a representative precipitate particle acquired from the segment of 0–0.7 cm.

For the control experiments, we performed reaction‐diffusion separations on a solution of 40 mM NdCl_3_ and 10 mM DyCl_3_ – not containing iron ions – using a gel loaded with 30 mM KOH as well as 10 mM Kdbp. The resulting precipitate patterns (Figure 3A) differed markedly from the Kdbp‐only experiments with identical feedstock compositions. Most notably, we observed the formation of periodic precipitation stripes, the characteristic feature of the Liesegang phenomenon. Note that the stripe patterns were not observed in experiments with Kdbp‐only or KOH‐only gel (Figure S10). The stripes consisted of crystals with a spherical core and multiple elongated spikes that grew outward in the radial direction (Figure 3B). While the crystals where localized within well‐defined regions, they were not in contact or aggregated together. The crystal size was of low polydispersity within each stripe and increased gradually from the solution‐gel interface to the end of the gel, about 17.9±4.7, 83.0±11.1, 147.2±17.8 μm at x=0.1, 0.4, and 1.0 cm, respectively (Figure 3B). These results were consistent with recent studies on material synthesis using reaction‐diffusion coupling, which reported a linear increase in particle size as a function of distance from the solution‐gel interface.^[38]^ Further along the tube, we observed a precipitate layer that extended 7.8 mm beyond the final Liesegang stripe. Closer examination showed that this layer also consisted of striped patterns.

To characterize these precipitate patterns, we averaged representative images along the tube radius and acquired a line profile that displayed the position and width of each stripe, both for the large‐scale pattern along the tube length (Figure 3C) as well as the micro‐scale pattern within the final precipitate layer at x > 1.4 cm (Figure 3D). The large‐scale pattern exhibited classical Liesegang features, with increasing spacing between the stripes such that the ratio of two consecutive stripe positions was nearly constant (Figure 3E). The empirical spacing coefficient (X_n+1_/X_n_) was ~1.2, comparable to other Liesegang precipitation patterns.[^24^, ^39^] This so‐called “spacing law” was complemented by the “time law”; the duration between two consecutive stripes increased, and the system followed square‐root dynamics (Figure S11). However, the stripes had a relatively constant width and hence did not obey the empirical “width law” (Figure S12). We also found increasing average particle sizes with increasing band number (Figure S13).

The micro‐scale pattern in the final precipitate layer showed qualitatively different features, with a nearly uniform spacing of 76.1±7.4 μm (Figure 3F). Recently, van Campenhout et al.^[40]^ observed equidistant silver dichromate stripes using a reaction‐diffusion system coupled to the mechanical response of a host gel. The result was ascribed to the ballistic, rather than diffusive, transport of the reactant species. We also observed similar equidistant stripes when extracting various transition metal hydroxides from battery feedstock solutions.^[18]^

Furthermore, Energy‐dispersive X‐ray spectroscopy(EDS) maps of the elemental distribution in the precipitates showed that all the particles contained both Nd and Dy (Figure 3G). ICP‐MS measurements of different segments indicated a slight Dy enrichment in the first half of the gel (up to 38.2 %) followed by increasing Nd content as the distance increased, up to a relative purity of 94.3 % towards the end of the gel (Figure S14). These results indicated that the presence of hydroxide (OH^−^) significantly affected the particle morphologies, induced band formation at two different scales, and diminished the enrichment of Dy in the segments closer to the solution‐gel interface.

### Separation of a Complex Feedstock with a Multi‐Reactant‐Loaded Gel

**Figure 4:**
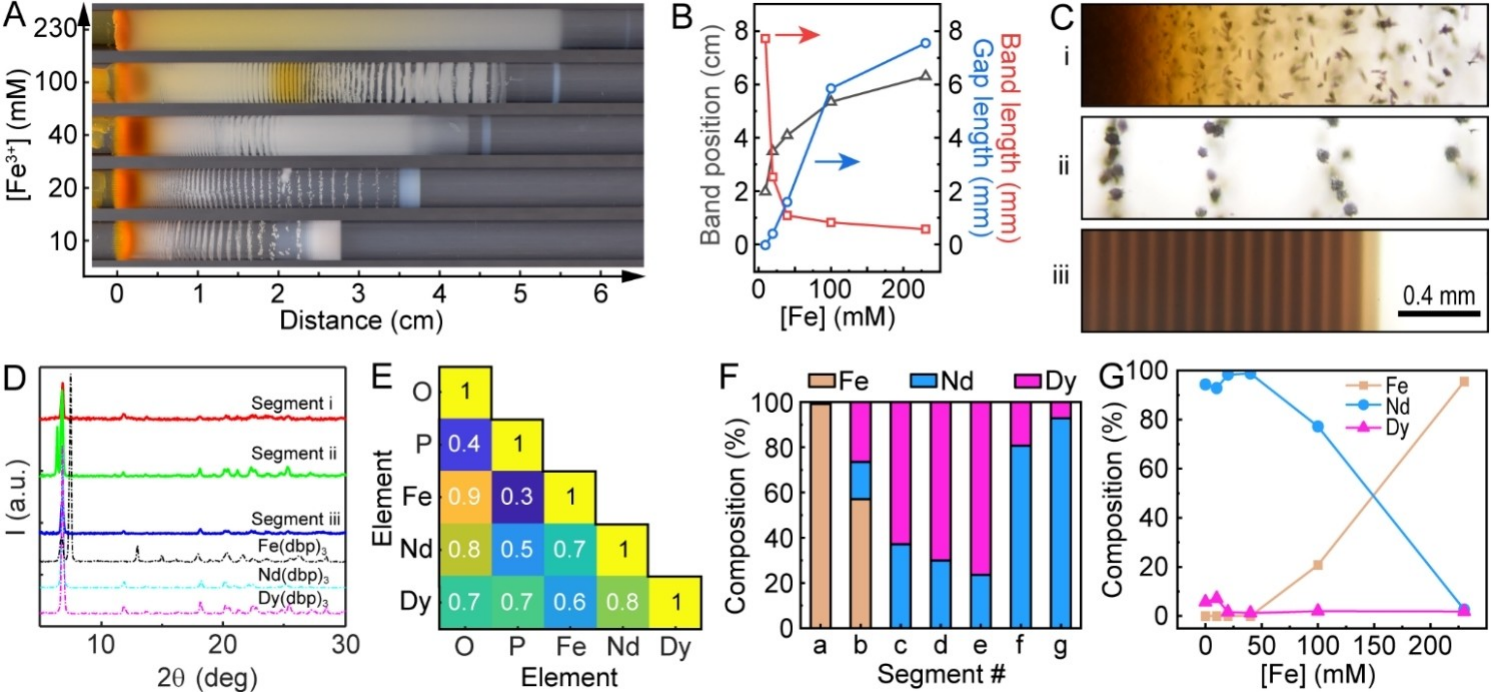
Reaction‐diffusion separations from feedstocks containing iron. (A) Photograph of the precipitate patterns for increasing Fe^3+^ concentrations. In these experiments, constant concentrations of Nd^3+^ (aq), Dy^3+^ (aq), dbp^−^ (gel), and OH^−^ (gel) were used, and the photograph was captured at *t*=5 days. (B) Measurements of the position, length, and gap of the leading band for each solution condition. (C) Optical micrographs of precipitates at the beginning, middle, and end of the gel labeled as segments (i), (ii), and (iii), respectively. (D) Powder XRD patterns of precipitates in different segments (solid lines) and pure precipitates for reference (dashed lines). (E) Correlation analysis of EDS maps for segment (i). (F) Molar composition along the precipitate from a mixed‐salt experiment measured by ICP‐MS. The Fe^3+^ concentration was 10 mM for C−F. (G) Molar composition at the end of the gel for different Fe^3+^ concentrations.

The final series of experiments involved validating reaction‐diffusion separations from model feedstock solutions that included iron, a typical component in recycled NdFeB magnets. We systematically varied the concentration of Fe^3+^ from 10–230 mM with a constant Nd^3+^ concentration of 40 mM and Dy^3+^ concentration of 10 mM as well as a constant gel composition of 10 mM Kdbp and 30 mM KOH (Movie S3). The resulting precipitate patterns are presented in Figure 4A. The total precipitate length increased from 2.8 cm at [Fe^3+^]=10 mM to 6.2 cm at [Fe^3+^]=230 mM. All of the solution conditions produced a yellowish‐orange layer near the solution‐gel interface, indicating the presence of iron phases such as α‐FeO(OH), as evident from Raman spectroscopy (Figure S15). Periodic precipitate stripes were observed in experiments with all Fe concentrations except 230 mM, and the spacing, width, color, and texture of these features varied. In addition, we noticed a leading band propagating at the front of each precipitate pattern, with increasing gap sizes and decreasing band lengths for increasing Fe^3+^ concentrations (Figure 4B). Moreover, we observed a smooth color gradient of orange to white for [Fe^3+^] 230 mM and a striking yellow region at 1.9–2.5 cm for [Fe^3+^] 100 mM, indicating the presence of Fe‐containing compounds. However, the white color in the neighboring regions did not guarantee an absence of Fe; some of the segments rapidly oxidized into an orange product upon exposure to air (Figure S16).

As a representative example, we characterized three segments of equal lengths of the precipitate pattern for [Fe^3+^]=10 mM. Optical micrographs showed individual rodlike particles in the first third (Figure 4C, i) similar to those observed in the Dy‐only experiment (Figure 1C). Further away from the solution‐gel interface, the individual rods formed bundles and spherical particles with rough surfaces (Figure 4C, ii). The final segment consisted of equidistant bands (Figure 4C, iii). Furthermore, the X‐ray diffraction (XRD) patterns of these three segments showed characteristic peaks[^17^, ^23^] of Nd(dbp)_3_ and/or Dy(dbp)_3_ that are nearly indistinguishable (Figure 4D). However, no characteristic peaks of Fe(dbp)_3_ or other iron (oxy)hydroxides were found, even in segment (i), where the orange color clearly indicated the presence of Fe. This result suggests that the Fe^3+^ precipitates were primarily in the form of amorphous hydroxides or oxides in this experiment. Accordingly, we collected EDS maps of these precipitates and analyzed the correlation of different elements. Our analysis showed that Fe was highly correlated with O but not with P for segment (i) (Figure 4E), indicating that Fe^3+^ preferably precipitates with OH^−^ rather than dbp^−^. Nd, Dy, and P were moderately correlated throughout different segments (Figure S17). We did not detect Fe signals in segment (iii) with EDS.

Additional characterization of the elemental composition was performed using ICP‐MS by measuring the concentrations of Fe, Nd, and Dy in parts‐per‐billion (ppb) in the digested samples. The relative percentages of these three components are reported in Figure 4F. We found a Fe‐pure (99.2 %) region at the solution‐gel interface (Figure 4F, a). The high concentration of Fe drastically decreased to 57.1 % in the next segment and was zero for the remaining segments. The Dy ratio increased to 76 % in the middle region (Figure 4F, e) and suddenly decreased, while the Nd content increased reaching 92.9 % at the end of the precipitate region (Figure 4F, g). These trends were consistent across a range of solution conditions. Specifically, high Nd purity (up to 98.8 %) was consistently found in the last part of the precipitate for a wide range of Fe^3+^ concentrations (0–40 mM). Further increases in Fe^3+^ concentration resulted in a high Fe content in the entire precipitates (Figures 4G and S18). These results demonstrate that reaction‐diffusion coupling may pre‐screen residual iron content in permanent magnet feedstocks, and further enrich and isolate the Dy and Nd components.

### Discussion of Separation Mechanisms and Broader Impact

Based on the experimental and simulated results, we evaluated the key properties of the system that were responsible for successful reaction‐diffusion separations. For this purpose, we calculated the Damköhler ratio; a dimensionless number that compares the relative timescales of the reaction kinetics and transport phenomena occurring in the system. For the case of reaction‐diffusion coupling, the ratio is given by:

(1) $$Da=\frac{kC^{n-1}\lambda^{2}}{2D}$$

where *k*, *D, C, n*, and *λ* represent the precipitation rate constant, ion diffusion coefficient, reactant concentration, reaction rate order, and lengthscale, respectively.[^41^, ^42^] At the relevant concentration (0.01 M) and lengthscale (10 cm), the Damköhler ratios are exceedingly small, *Da_Nd_* =9.8×10^−16^ and *Da_Dy_* =4.6×10^−13^, indicating that the system is reaction‐limited. The Damköhler ratios are ≪1 for extended lengthscales of several meters, suggesting that scaling up the reactor dimensions would be favorable for larger throughput separations. This result also demonstrates why reaction‐diffusion separation was highly successful for this particular feedstock: in a reaction‐limited regime, the vast difference between *k_Nd_* and *k_Dy_* was the key parameter in controlling the spatial distribution of the corresponding products.

Despite several anticipated limitations, the reaction‐diffusion model was highly successful in predicting the spatial and temporal evolution of Dy and Nd precipitates in the reaction medium. Specifically, the precipitation rate term is a simplified representation that combines multiple phenomena, such as nucleation, growth, and aggregation, with complex dependencies on solution conditions beyond the consideration of continuum models. For example, nucleation rates are notoriously difficult to characterize, even in well‐defined bulk media,^[43]^ let alone under gradients of supersaturation conditions. At far‐from‐equilibrium conditions, the early crystallization stages follow unexpected pathways that cannot be predicted from equilibrium phase diagrams.[^44^, ^45^] Another key phenomenon is crystal growth, the rate of which also depends on atomic‐scale factors such as ion and surface (de)hydration energies.^[46]^ In addition, the ability of newly formed particles to aggregate upon nucleation in the early growth stages changes the distribution of products.[^47^, ^48^] All of these phenomena, and the coupling thereof, are lumped into a single reaction rate term, which can qualitatively predict the distribution of the products and the separation efficiency, but it is not expected to capture the underlying details at the atomistic scale.

Gel media present further challenges in addressing the role of confinement,^[49]^ heterogeneous nucleation,[^50^, ^51^, ^52^] and interfacial solution structure[^53^, ^54^] which may drastically alter the particle growth landscape. Accordingly, consolidating all these competing and coupled effects into a single rate term – although highly effective in the present study – would be more challenging for complex feedstocks with non‐ideal chemical compositions. For example, the presence of competing ions in multicomponent solutions alters the nucleation rates in non‐intuitive ways[^55^, ^56^] and incorporates impurities within a primary phase,^[57]^ among other effects.^[58]^ It requires the development of multiscale models that faithfully encompass the atomistic details of the structure and interactions in multicomponent electrolytes. In addition to tuning the gel properties, the precipitate formation could also be fine‐tuned by applying external fields (thermal, electric, magnetic) to optimize the distribution of the high‐purity segments.

## Conclusions

In this study, we demonstrated reaction‐diffusion separations as a viable energy‐efficient approach for selectively recovering critical materials from unconventional feedstock solutions, namely permanent magnets that are typically present in spent consumer electronics. Starting with a model mixture of dysprosium(III) and neodymium(III) chloride, we observed the precipitation of a dysprosium‐enriched phase followed by a relatively pure neodymium phase along the length of the reaction medium. These experimental results were accurately reproduced in reaction‐diffusion simulations. Moreover, we observed the pre‐screening of up to 40 mM of iron by precipitating an oxide phase near the solution‐gel interface. This result validated the robustness of reaction‐diffusion coupling for handling feedstock complexity.

One potential advantage of reaction‐diffusion separations is the chemical versatility and applicability to a variety of feedstock conditions. Other cutting‐edge separation methods are based on optimizing atomic‐scale properties, such as ligand binding energies,[^59^, ^60^] membrane pore sizes,^[61]^ ionic liquid interactions,[^62^, ^63^] or equilibrium distributions across various solvents.^[64]^ The challenge with tailoring these properties is that confounding factors in multicomponent solutions and non‐ideal conditions may drastically compromise the separation efficiency.[^65^, ^66^] In comparison, the reaction‐diffusion approach is based on simple precipitation reactions using commodity chemicals that are already deployed in separation industries. Separations are achieved by leveraging subtle differences in the intrinsic physicochemical properties of the target ions, namely the diffusion and precipitation rates, which are more robust to feedstock variability. For example, we recently reported the extraction of an almost pure manganese phase from model solutions of recycled batteries using sodium hydroxide, with robust results from five candidate cathode materials.^[18]^

Going forward, several challenges need to be addressed to translate this proof‐of‐concept study into applied technologies. Firstly, the recovery of the solid products from the gel material needs to be more efficient. Secondly, the timescales for separations need to be accelerated – although this issue is partially mitigated by the fact that the system is passively operated. We anticipate that applying hydraulic pressure,^[73]^ external electromagnetic fields, or other strategies from the chromatography industry may offer solutions to these challenges, which are beyond the scope of the current study. Future work will thus aim to enhance the capacity, throughput, and efficiency of reaction‐diffusion separations beyond the laboratory scale.

## Supporting Information

The authors have cited additional references within the Supporting Information (Ref. [17,46]).

## Author Contributions

Q.W. performed the experiments, data analysis, and contributed to the study design. Y.F. performed the reaction‐diffusion simulations. Z.X. supervised the reaction‐diffusion simulations. D.S. performed the cDFT simulations. M.L.S. supervised the cDFT simulations. P.J.B., A.R., and E.A.M. performed the X‐ray imaging experiments. B.B. supervised the X‐ray imaging experiments. G.E.J. provided useful input on the current landscape of chemical separations and REE feedstocks. E.N. designed and supervised the study.

## Conflict of Interests

The authors declare no conflict of interest.

1

## Supporting information

As a service to our authors and readers, this journal provides supporting information supplied by the authors. Such materials are peer reviewed and may be re‐organized for online delivery, but are not copy‐edited or typeset. Technical support issues arising from supporting information (other than missing files) should be addressed to the authors.

Supporting Information

Supporting Information

Supporting Information

Supporting Information

## Data Availability

The data that support the findings of this study are available in the supplementary material of this article.
