# Supplementary material for: Selective Recovery of Critical Minerals from Simulated Electronic Wastes Via Reaction‐Diffusion Coupling
Source: ChemSusChem. 2025 Feb 18;18(10):e202402372. doi: 10.1002/cssc.202402372 (PMC12094141; doi:10.1002/cssc.202402372)
Supplement: Supplementary file 1 — Supporting Information [file CSSC-18-e202402372-s001.pdf]

ChemSusChem

Supporting Information

Selective Recovery of Critical Minerals from Simulated Electronic Wastes Via Reaction-Diffusion Coupling

Qingpu Wang, Yucheng Fu, Erin A. Miller, Duo Song, Philip J. Brahana, Andrew Ritchhart, Zhijie Xu, Grant E. Johnson, Bhuvnesh Bharti, Maria L. Sushko, and Elias Nakouzi*

Supporting Information

Selective Recovery of Critical Minerals from Simulated Electronic Wastes via Reaction-Diffusion Coupling

Qingpu Wang,[a] Yucheng Fu,[b] Erin A. Miller,[c] Duo Song,[b] Philip J. Brahana,[d] Andrew Ritchhart,[b] Zhijie Xu,[b] Grant E. Johnson,[b] Bhuvnesh Bharti,[d] Maria L. Sushko,[b] and Elias Nakouzi*[b]

[a] Physical and Computational Sciences Directorate, Pacific Northwest National Laboratory, Seattle, Washington 98109, United States

[b] Physical and Computational Sciences Directorate, Pacific Northwest National Laboratory, Richland, Washington 99354, United States

[c] National Security Directorate, Pacific Northwest National Laboratory, Richland, Washington 99352, United States

[d] Cain Department of Chemical Engineering, Louisiana State University, Baton Rouge, Louisiana 70803, United States

*Email: elias.nakouzi@pnnl.gov

Table of Contents

1. Experimental Methods and Modeling Details

2. Supporting Figures and Tables

3. Supporting Movies

4. Supporting References

1. Experimental Methods and Modeling Details

Chemicals and Materials. Agarose (type I, low electroendosmosis [EEO], Sigma-Aldrich), dibutyl phosphate (Hdbp, $[CH_3[CH_2]_3]_2HPO_4$, 97%, Thermo Scientific Chemicals), potassium hydroxide, (KOH, Fisher Scientific), neodymium chloride hydrate ($NdCl_3{\cdot}H_2O$, 99.9% rare earth oxide [REO], Thermo Scientific Chemicals), dysprosium chloride($DyCl_3$, anhydrous, 99.9% Dy, Fisher Chemical), and nitric acid (HNO_3, Trace Metal grade, Fisher Chemical) were used as received. All solutions were prepared with deionized water (resistivity, 18.2 MΩ cm) filtered by a water purification system (Milli-Q IQ 7000).

Gel Preparation. In a typical experiment, 0.2 g of agarose powder were dissolved in 10 mL of DI water in a hot water bath at about 90 °C. Then 2.5 mL of the hot agarose solution (2 wt%) were mixed with a preheated 1.0 M Kdbp (and/or 1.0 M KOH) solution and hot DI water to yield the desired reactant concentration of 10 mM Kdbp (and/or 30 mM KOH) with 0.5 wt% agarose. The mixed liquids were stirred in the hot water bath until homogeneous, subsequently transferred to glass tubes (inner diameter 4.2 mm, outer diameter 5 mm, length 178 mm), and cooled at room temperature for further use.

Diffusion Experiments and X-ray Imaging. 0.5 mL of 1.0 M $NdCl_3$ or $DyCl_3$ were added to a cuvette containing agarose gel (0.5 wt%). X-ray radiography was used to track the diffusion of REEs into the gel. Cuvettes were placed approximately 10 cm in front of a digital X-ray detector (Shad-o-box 6k HS, 49-micron pixels). The detector was approximately 2 m from the source (Comet MXR-160HP/11, operated with a 1 mm focal spot). Images were acquired with an X-ray endpoint energy of 160 kV and a current of 2 mA, and each image was averaged from 120 frames (0.5 seconds exposure time each). All images were flat and dark corrected, such that the resulting intensities represented the fraction of radiation transmitted. The ion concentration was determined from the image contrast as

$$C = -\ln(int) \qquad \text{(Eq. S1)}$$

Where *C* and *int* represent the ion concentration and image intensity, respectively, and normalized against the initial image contrast for the bulk solution at 1.0 M.

Precipitation Experiments. Solutions of individual or mixed salts of $FeCl_3$, $NdCl_3$, and $DyCl_3$ were added in the glass tubes containing the reactant-loaded gel with a gel-to-solution volume ratio of 3 (solution length 40 mm, gel length 120 mm). All experiments were performed at room temperature for about 5 days. The progress of precipitate formation was recorded using a digital single-lens reflex (DSLR) camera (Nikon D5500) controlled by a personal computer (PC) and digiCamControl software. The collected image data were analyzed using custom scripts in MATLAB (MathWorks, R2022b).

Characterization. At the end of the time-lapse recording, micrographs of the samples were collected using an inverted optical microscope (AmScope IN480T) and a digital camera (Nikon D5500). Then, the precipitate samples were extracted from the glass tubes, washed in hot water, and air-dried for further characterization using powder-X-ray diffraction (XRD, Bruker D8 Discover Microfocus diffractometer), Raman microscope (Renishaw InVia, excitation wavelength, 785 nm), scanning electron microscopy (SEM, FEI Sirion XL30), and energy-dispersive X-ray spectroscopy (EDS, Oxford Instruments). In addition, the precipitate samples were digested in concentrated nitric acid (HNO_3,70 wt%), and their elemental composition was measured by inductively coupled plasma mass spectrometry (ICP-MS, PerkinElmer NexION 2000).

Time-space plots. We constructed the time-space plots using MATLAB. We obtained a color profile from each frame by averaging the image along the tube's radius. These color profiles were then stacked chronologically to the time-space plot.

Reaction-Diffusion Simulations. The diffusion and precipitation of Nd and Dy were simulated and predicted based on the transient reaction-diffusion model described as follows:

$$\frac{\partial c_i}{\partial t} = D_i \frac{\partial^2 c_i}{\partial x^2} + R_i \qquad \text{(Eq. S2)}$$

where c_i is the concentration, D_i is the diffusion coefficient, and R_i is the reaction rate of the ith species, respectively. The tracked species included $i \in \{Nd^{3+}, Dy^{3+}, dbp^{-}\}$. The source term R_i stands for the species consumption rate related to the precipitation of solid species j ($Nd(dbp)_{3(s)}$ or $Dy(dbp)_{3(s)}$) as:

$$R_i = -\sum_j \gamma_{i,j} R'_j \qquad \text{(Eq. S3)}$$

where $\gamma_{i,j}$ is the number of moles of the ionic species i in the precipitate j. The precipitation rate R'_j was calculated as

$$R'_j = k_j \left[\prod_i c_i^{\gamma_{i,j}} - K_{sp,j} \right] \qquad \text{(Eq. S4)}$$

Here, k_j stands for the precipitation rate and $K_{sp,j}$ is the solubility product of the precipitate j. The model was solved in a one-dimensional format by assuming the variation of those related species in the experiment occurs along the tube length direction. The simulations were implemented and carried out using the COMSOL Multiphysics software®.[1]

The setup for our numerical reaction-diffusion model is illustrated in Figure S7, which consists of two connected domains: a salt solution domain and a gel domain. The model assumes variation in species concentration along the tube's length in the x-direction, as indicated in the figure. The simulation was conducted in a 1D format, with concentration variations only along the x-axis. The top boundary of the solution domain was treated as a Neumann boundary with no flux of species. The bottom boundary of the gel domain was set to a zero species concentration condition, as it was sufficiently distant from the solution domain. The interface between the solution and gel domains enforces continuity in both concentration and flux. In the initial step of the study, the goal was to measure the diffusion coefficient of individual salts (as shown in Figure 2, Step 1), where the setup consisted of a salt solution domain with a length of L_{sol} = 0.5 cm followed by a gel domain with a length of L_{gel} = 5.0 cm. This configuration resulted in a mesh discretization of 550 elements. For the subsequent steps, which involved reaction-diffusion processes for both individual and mixed salts, the salt solution domain had a length of 4 cm, followed by a 12 cm gel domain. These setups used a total of 10,667 mesh elements. The mesh grid was refined at the edges of the domains and at the solution/gel interface to accurately capture any sharp changes in these regions.

The parameters used in reaction-diffusion model (Eq. 3) are summarized in Table S1. The diffusion coefficients of Nd^{3+} and Dy^{3+} were determined by comparing the numerical simulation results against the experimental diffusion data, as reported in Figure 2A. Figures S6A-C illustrate the process of determining the diffusion coefficient of the Nd^{3+} ion. In the Figure S6A, the logarithmic scale of image intensity, $-\ln(int)$, within the gel domain was plotted for three different measurement times. The ion concentrations were then derived from these image intensity distributions by normalizing the maximum intensity to 1.0 M, which was located near the solution/gel interface at the beginning of the study, as depicted in Figure S6B. During the numerical simulations, a wide range of diffusion coefficients were tested to generate corresponding concentration distribution curves. These simulated concentration curves were then compared with the experimental data to identify the best fit. Figure S6C shows the mean absolute error when comparing the various numerical simulations with the experimental data. The diffusion coefficient of Nd^{3+} was determined by locating the point of minimum mean absolute error on this curve. Those procedures were also employed to determine the diffusion coefficient of Dy^{3+}, with the details provided in Figures S6D-F. The precipitation rates k_{Nd} and k_{Dy} were determined using a similar parameter estimation approach. A

broad range of values for k was tested to generate the precipitate concentration profiles, which were then compared to the experimental data (Figure 2, Step 2). This comparison allowed for the identification of the best-fitting k values used in the reaction-diffusion models.

The diffusion coefficient of dbp^- was calculated using the Stokes-Einstein equation, which assumes that the diffusion coefficient of a molecule increases proportionally to the inverse of its radius, a factor correlated with the ion's molecular weight. Based on the relationship between molecular weight and diffusion coefficient reported by Miyamoto and Shimono[2], the diffusion coefficient was fitted to be 6.92×10^{-10} m^2/s for dbp^- (Figure S8).

Simulations of Ion Diffusivity. Atomistic-to-mesoscale simulations of ion diffusivity in electrolyte solutions were performed using Poisson-Nernst-Plank formalism coupled with classical Density Functional Theory (PNP-cDFT) the ion flux, J_i, was calculated under the steady state conditions ($\nabla J_i = 0$) as[3]

$$-J_i = D_i(r)\left[\nabla\rho_i + \frac{1}{k_B T}\rho_i\left(q_i e\nabla\varphi + \nabla\mu_i^{id}(r) + \nabla\mu_i^{ex}(r)\right)\right] \qquad \text{(Eq. S5)}$$

$$-\nabla(\varepsilon(r)\nabla\varphi) = 4\pi\left(\rho_f(r) + e\sum_i q_i\,\rho_i\right) \qquad \text{(Eq. S6)}$$

In these equations $D_i(r)$ and ρ_i are the diffusion coefficients and densities, respectively, of all the species, φ is the electrostatic potential, m^{id} and m^{ex} are the ideal and excess chemical potential, respectively, $\rho_f(r)$ is the fixed charge density in the system (if any), $k_B T$ is the thermal energy, and e is the electron charge. Index "i" denotes mobile species type, e.g., ions and solvent molecules. cDFT was used for the evaluation of the chemical potentials of all species and the total free energy.[4]

2. Supporting Figures

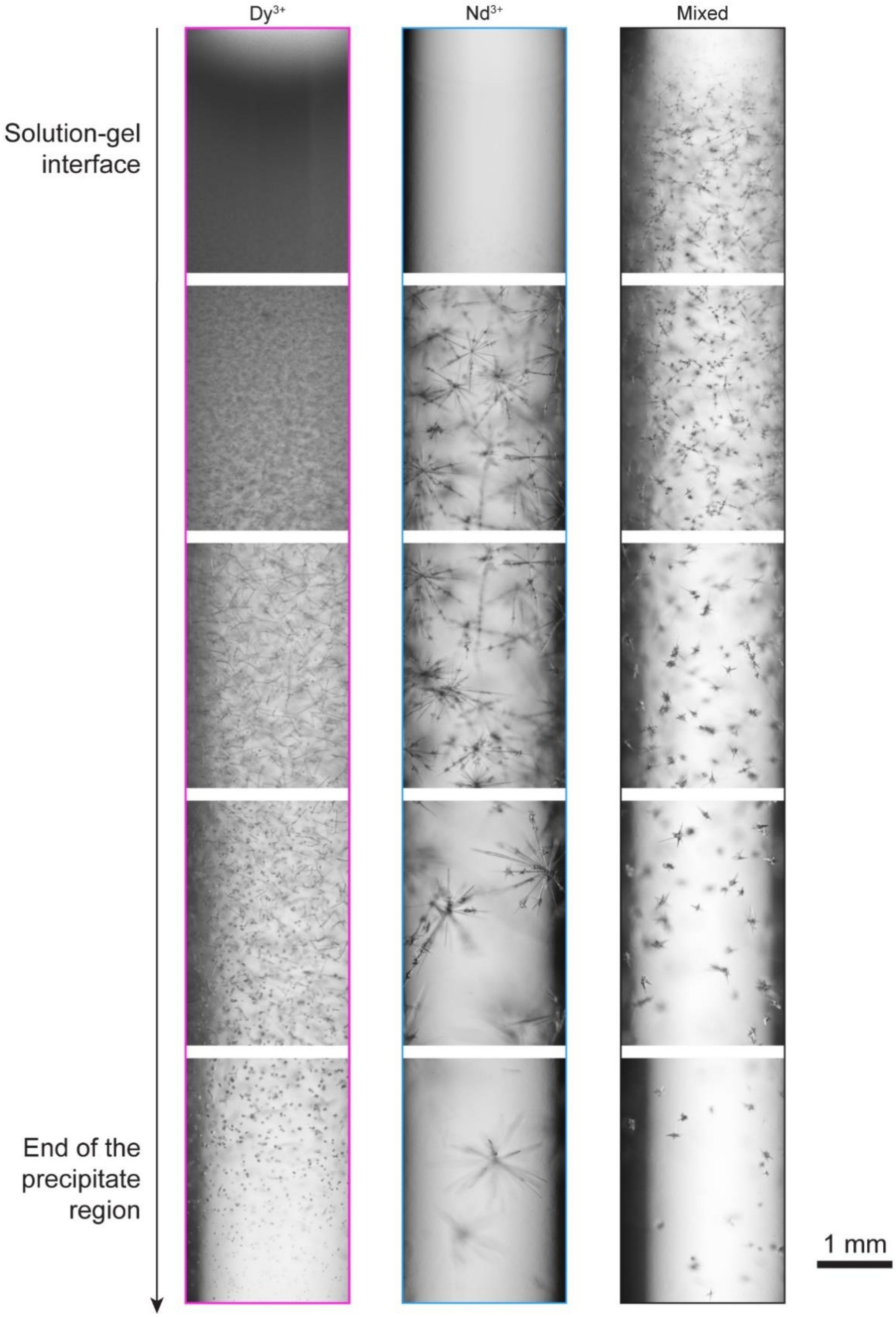

Figure S1. Micrographs at different locations along the gel for Dy-only (magenta), Nd-only (cyan), and mixed-salt (black) experiments. The five images in each column approximately correspond to the 5%, 20%, 50%, 80%, and 95% of the entire precipitate length. All images are at the same scale.

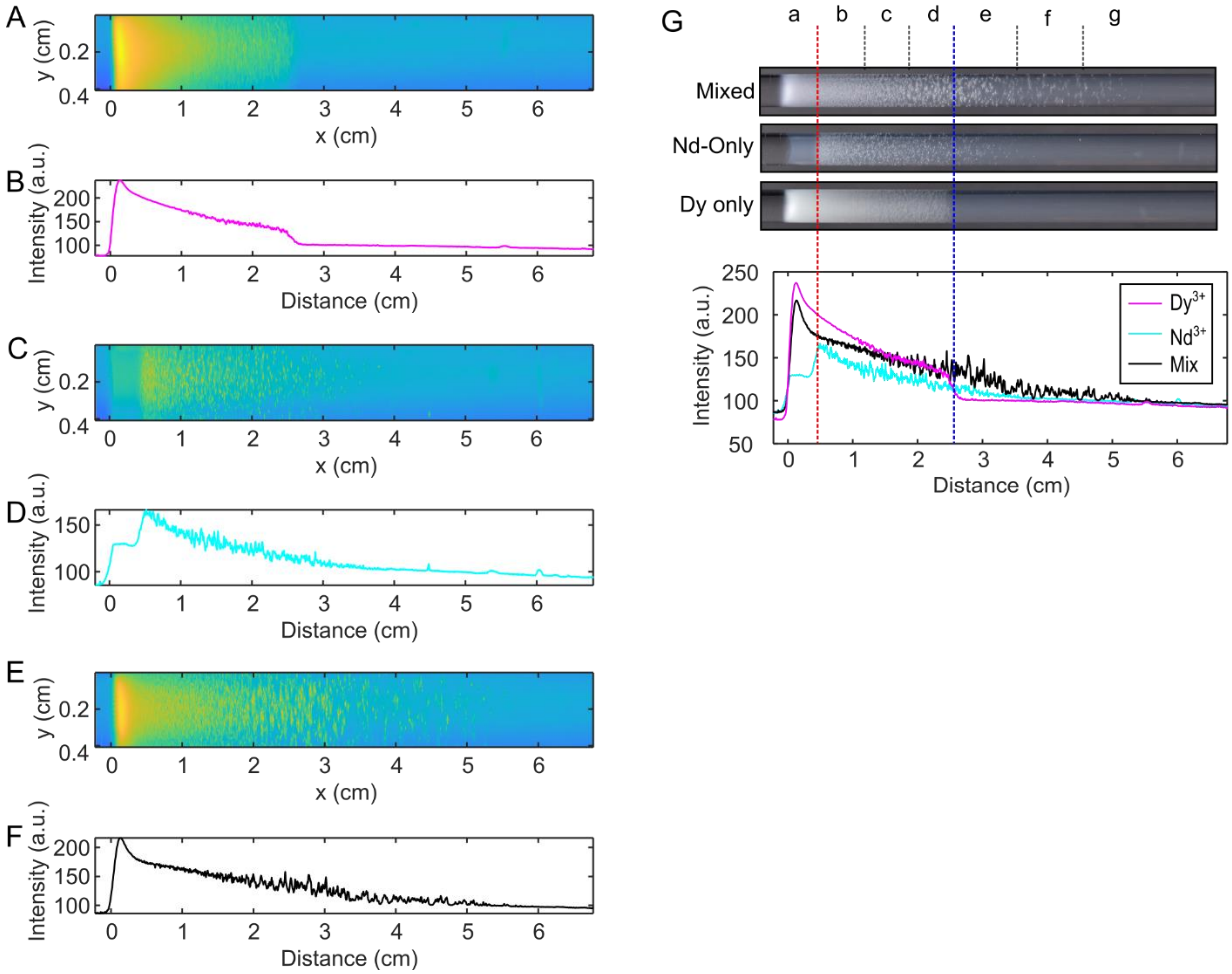

Figure S2. (A,C,E) False-colored images and (B,D,F) line profiles of the individual and mixed salt experiments shown in Figure 1B. (A,B) Dy-only, (C,D), Nd-only, and (E,F) mixed salt. (G) Segmentation of the mixed-salt experiment was informed by the individual salt experiments. The red line indicates the border of segments a and b which was the translucent layer near the solution-gel interface in the Nd-only experiment. The blue line indicates the border of segments d and e which marked the end of the precipitate region in the Dy-only experiment.

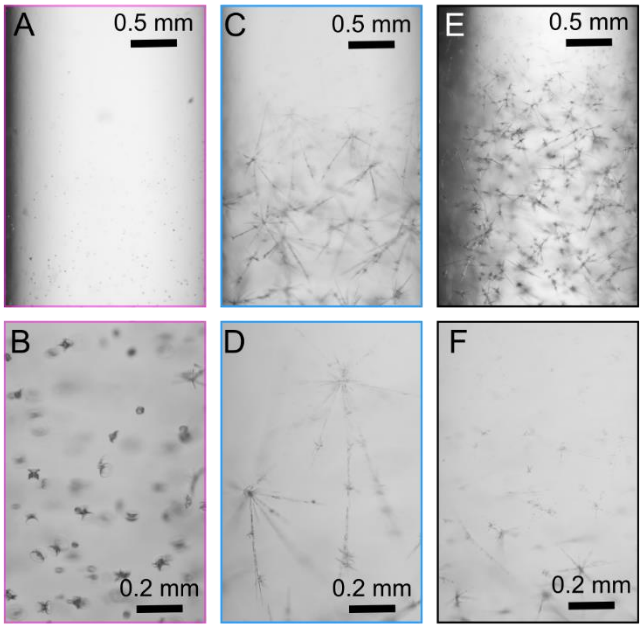

Figure S3. Micrographs near the solution-gel interface for Dy-only (A,B), Nd-only (C,D) and mixed-salt (E,F) experiments. A-E were captured at the same scale with a 4X objective lens. B-F were captured at the same scale a 10X objective lens.

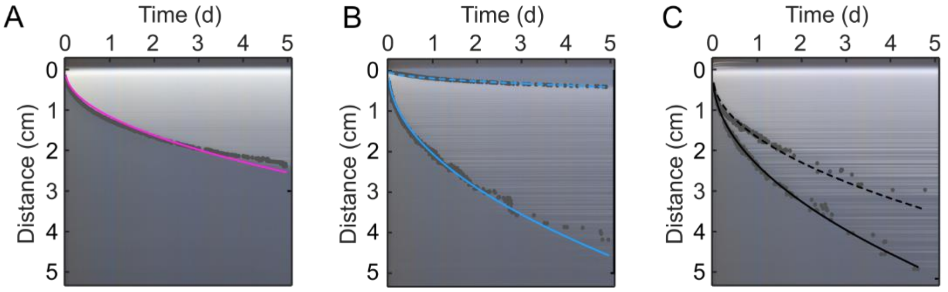

Figure S4. Time-space plots shown in Figure 1E with superimposed growth fronts for Dy-only (A), Nd-only (B), and mixed-salt (C) experiments.

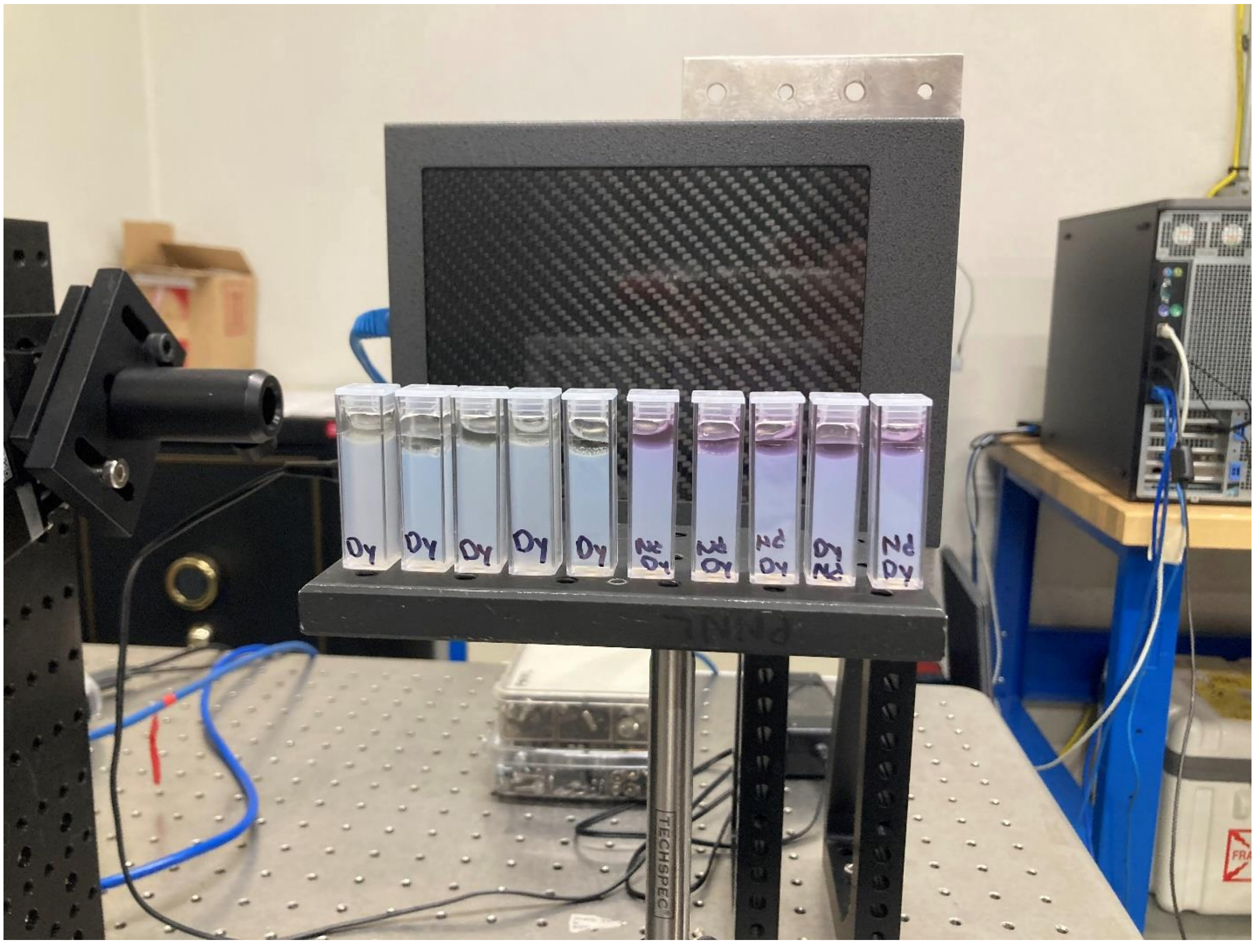

Figure S5. Photograph of the phase-contrast X-ray imaging setup.

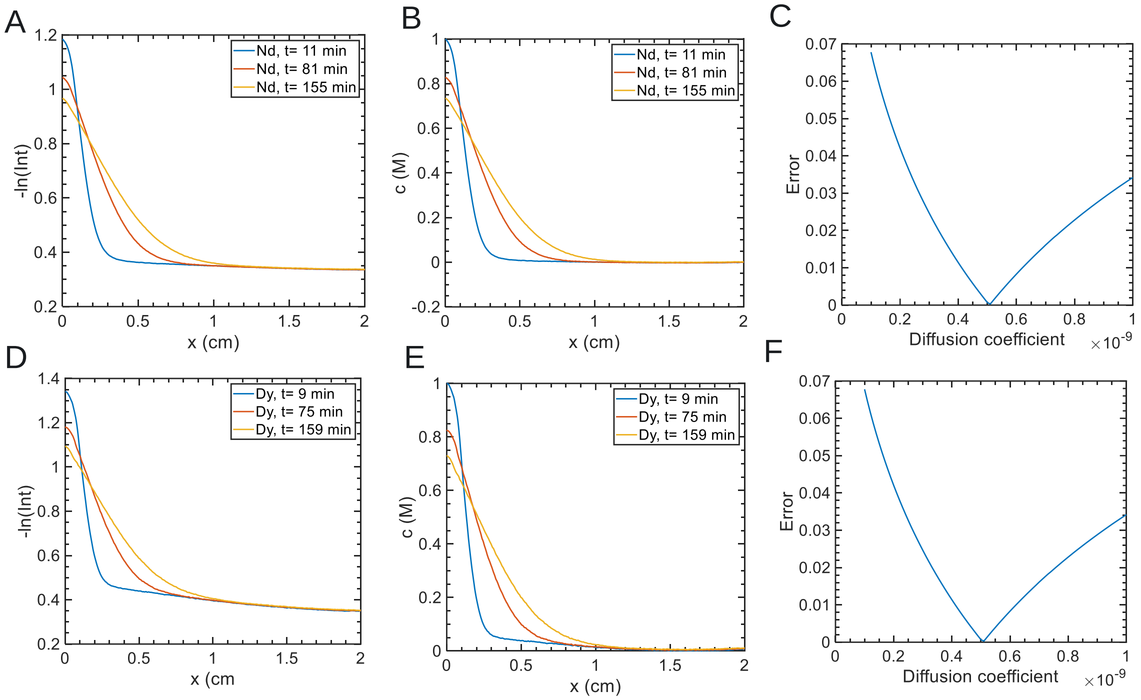

Figure S6. Steps for recovering the diffusion coefficients for Nd^{3+} (A-C) and Dy^{3+} (D-F) using numerical simulations compared to experimental data. Column 1 shows the image intensity distribution at three different time points at the logarithmic scale. Column 2 displays the corresponding concentration distributions across the gel domain. Column 3 illustrates the optimization process based on the calculations of the mean absolute error by comparing concentration profiles from the experiment with those from numerical simulations, using a wide range of diffusion coefficients.

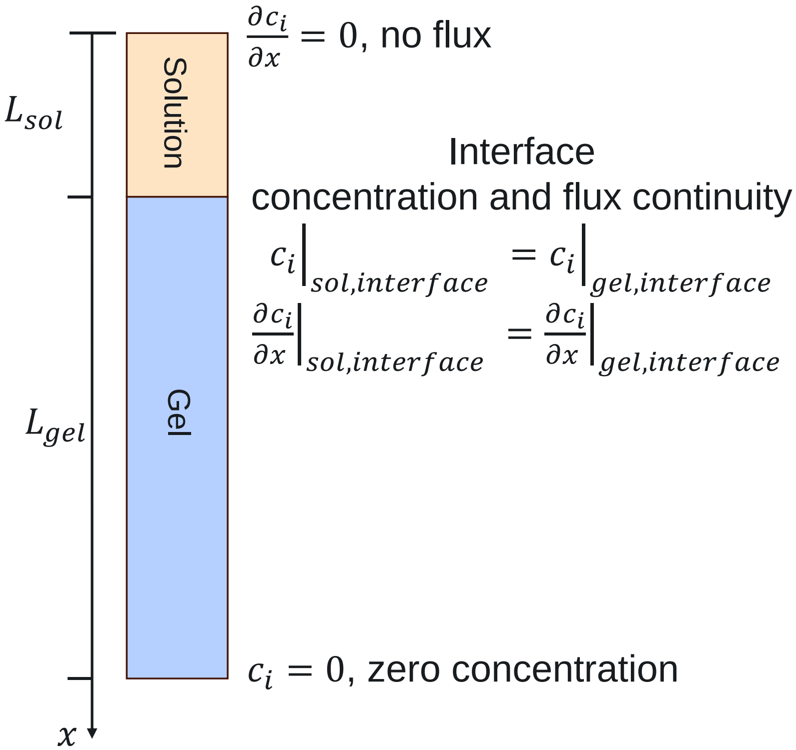

Figure S7. Sketch of the numerical model setup and boundary conditions for studying ion reaction-diffusion process. The model includes two connected domains: a solution domain and a gel domain, with lengths of L_{sol} and L_{gel}, respectively.

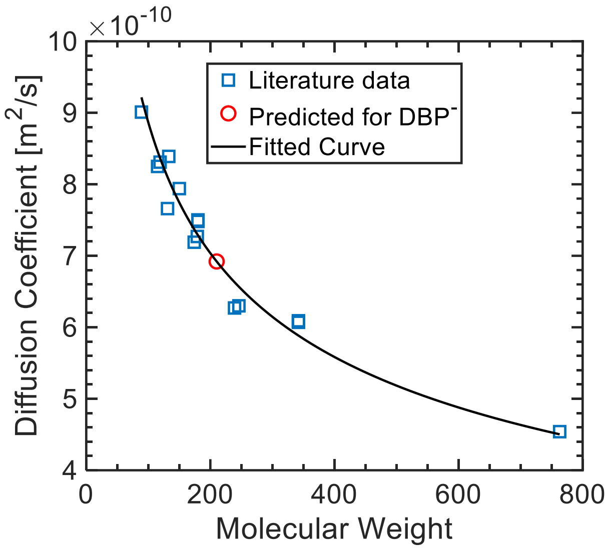

Figure S8. Estimation of the diffusion coefficient based on the molecular weight and Stokes-Einstein equation suggested by Miyamoto and Shimono[2].

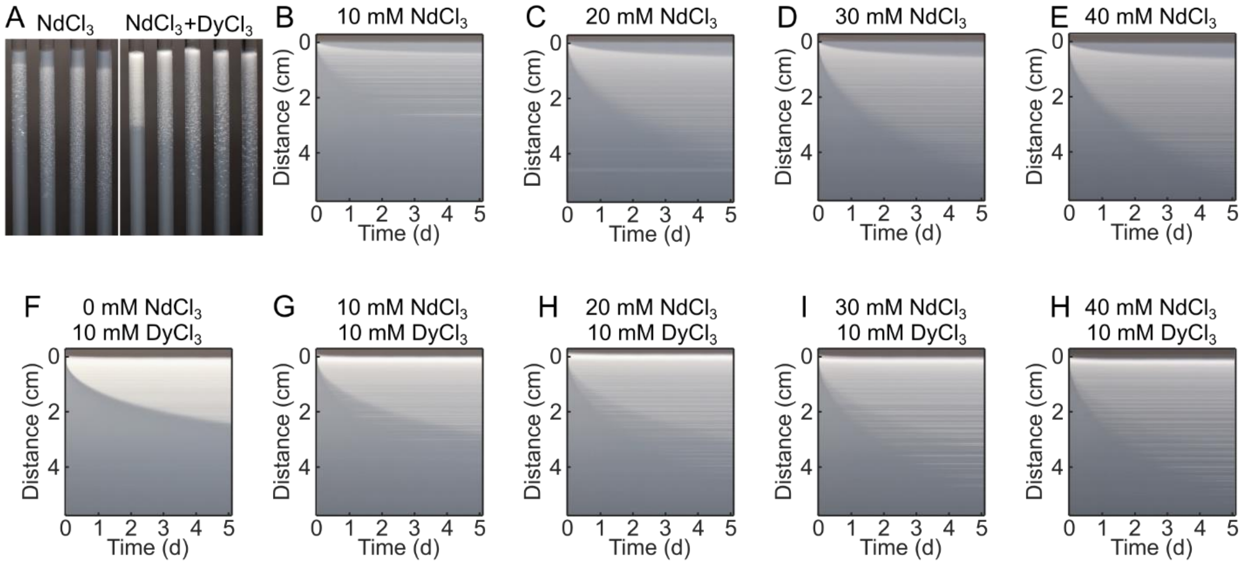

Figure S9. (A) Photographs of the precipitation patterns for Nd-only (left) and mixed salt (right) experiments. For the left panel, the $NdCl_3$ concentrations in the solutions were 10, 20, 30, and 40 mM, respectively from left to right. For the right panel, the solutions contained a constant $DyCl_3$ concentration of 10 mM and increasing concentrations of $NdCl_3$ from 0 to 40 mM. (B-H) Times-space plots of Nd-only (B-E) and mixed-salt (F-H) experiments with systematic concentration variations.

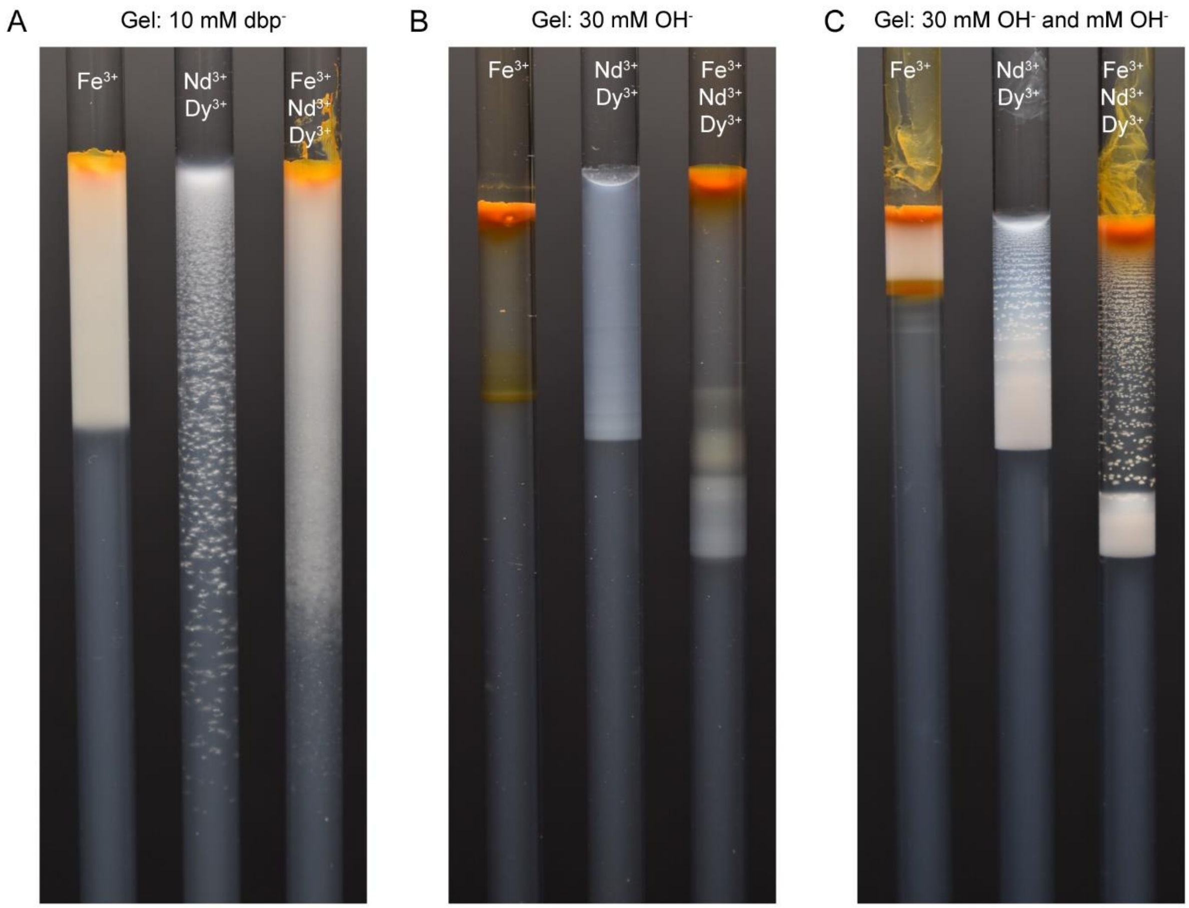

Figure S10. Photographs of control experiments with individual dbp^- (A), OH^- (B) and mixed reactants (C) in the gel. For each gel group, the salt solution contained (i) 10 mM Fe^{3+}, (ii) 40 mM Nd^{3+}, and 10 mM Dy^{3+}, and (iii) 10 mM Fe^{3+}, 40 mM Nd^{3+}, and 10 mM Dy^{3+}, respectively. The tube diameter was 5 mm for all. Photos were collected after 7 days of reaction.

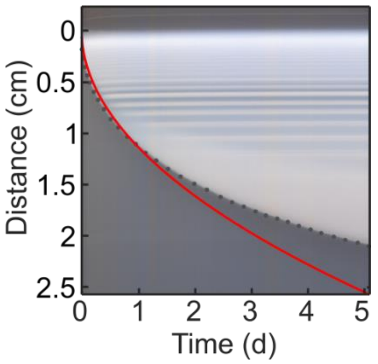

Figure S11. Time-space plot of the mixed-salt experiment with a gel containing two precipitation reagents. The sale solution contained 40 mM Nd^{3+} and 10 mM Dy^{3+}. The gel contained 10 mM dbp^{-} and 30 mM OH^{-}. The red curve is a square-root fit and deviates after the formation of the last periodic bands at x = 1.4 cm.

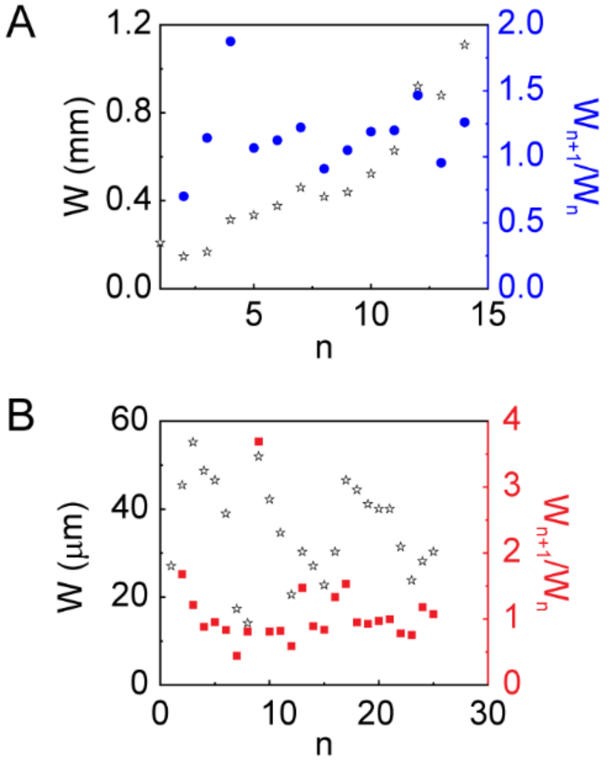

Figure S12. Width measurements for the large-scale (A) and small-scale (B) bands. Our results showed that the ratio of two consecutive bands varied for both the large- and small-scale band groups. For the large-scale bands, the variations are possibly due to the inhomogeneous distribution of particles in each band, whereas the conventional Liesegang bands contain uniform layers of precipitates.

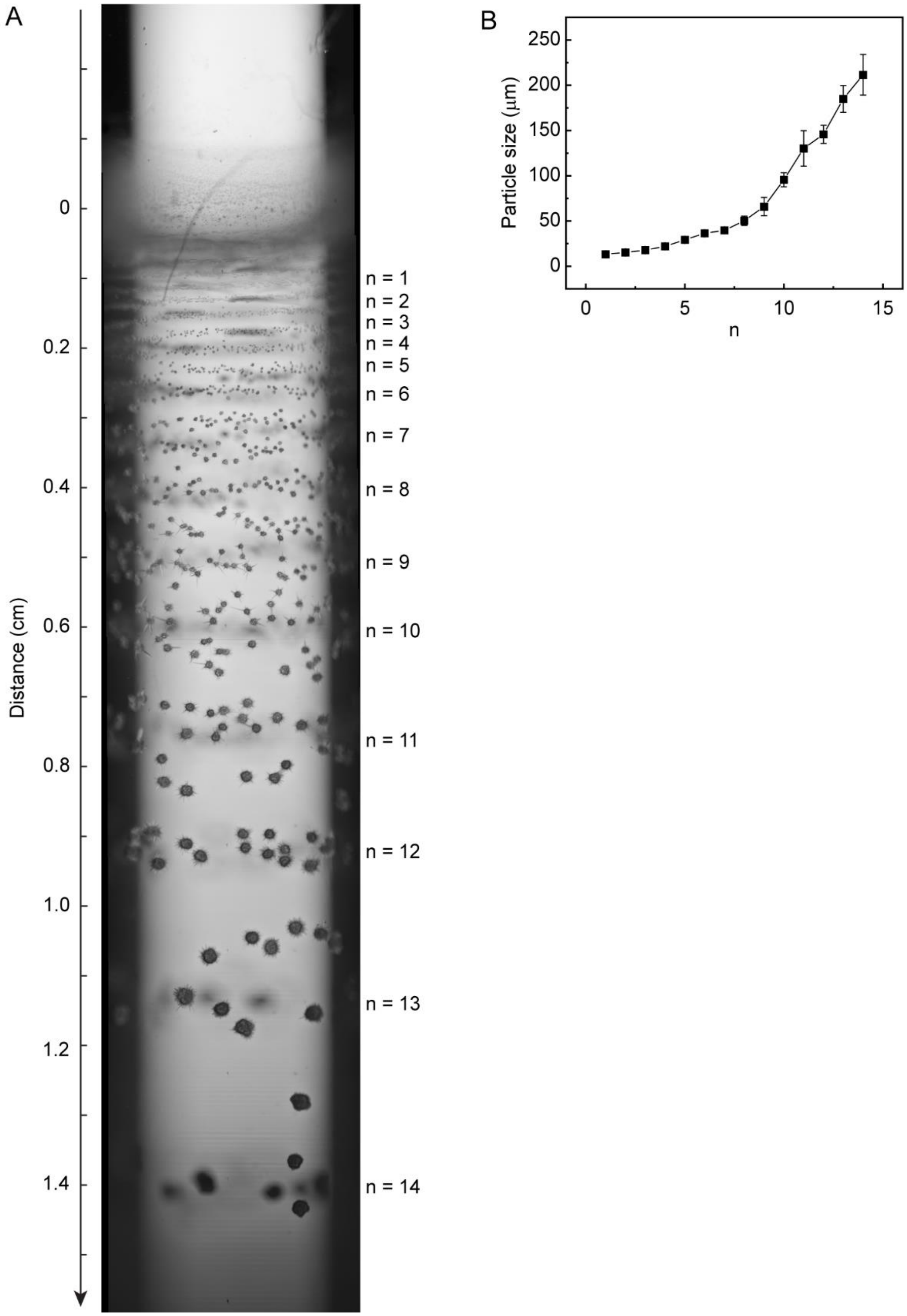

Figure S13. (A) Stitched micrographs of the precipitate pattern shown in Figure 3A. (B) Particle size as a function of band number for the large-scale band group. Note that the micro-scale bands overlapped with the last two large-scale bands (n=13 and 14).

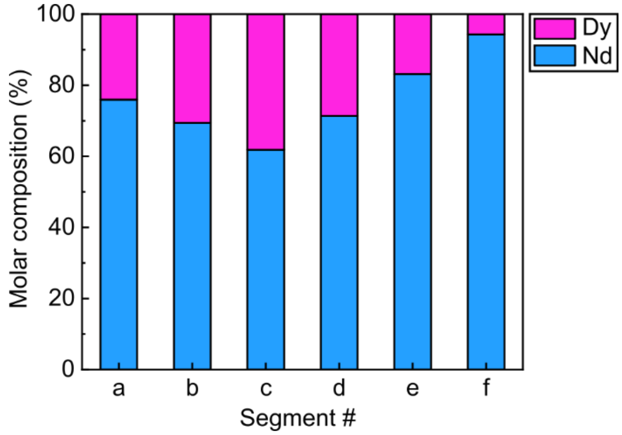

Figure S14. Molar composition along the precipitate pattern measured by ICP-MS. The samples were obtained from a mixed-salt experiment shown in Figure 3A.

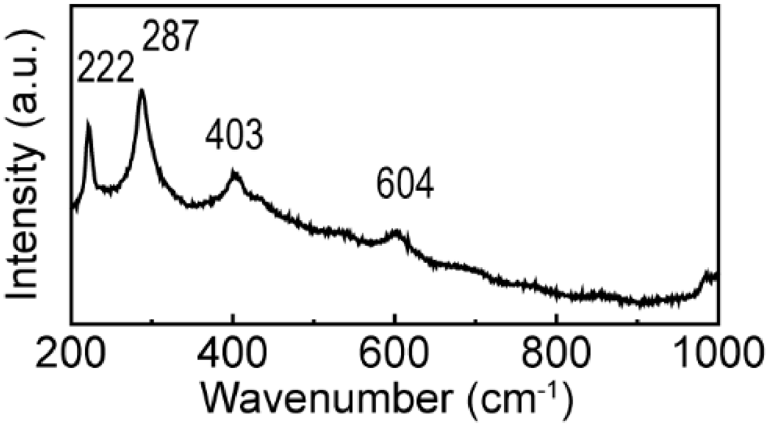

Figure S15. Raman spectrum of a segment sampled at the solution-gel interface with [Fe^{3+}] = 10 mM. The sharp peaks at 222, 287, and 403 cm^{-1} indicate a possible match with goethite a-FeO(OH).[5]

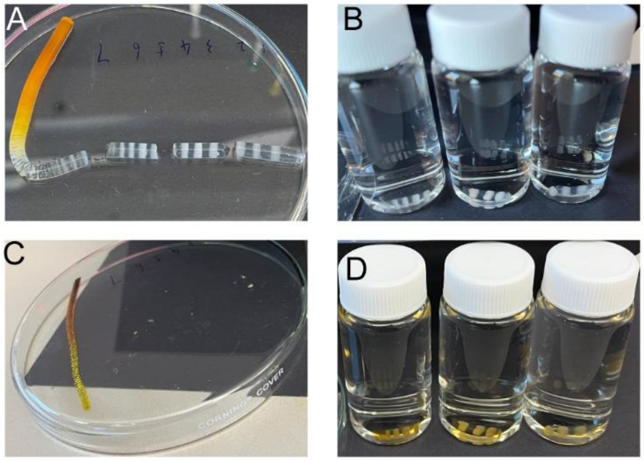

Figure S16. Photographs of precipitate samples before (A, B) and after 5 days exposed to air (C) and submerged in DI water (D).

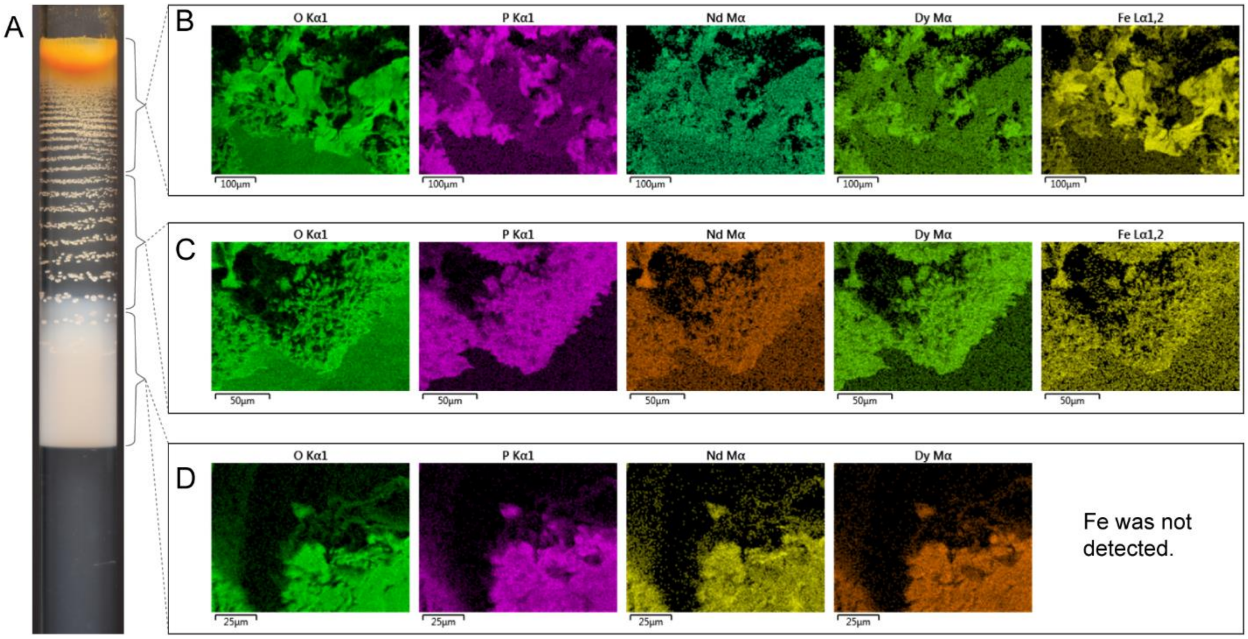

Figure S17. (A) Photograph of segment positions and (B-D) EDS maps of three segmented samples for $[Fe^{3+}]$ = 10 mM. The images in B were used for the correlation analysis shown in Figure 4E. The analysis was performed by converting the false-colored EDS maps to grayscale, cropping them to a region of interest, and computing the pairwise correlation coefficients using MATLAB.

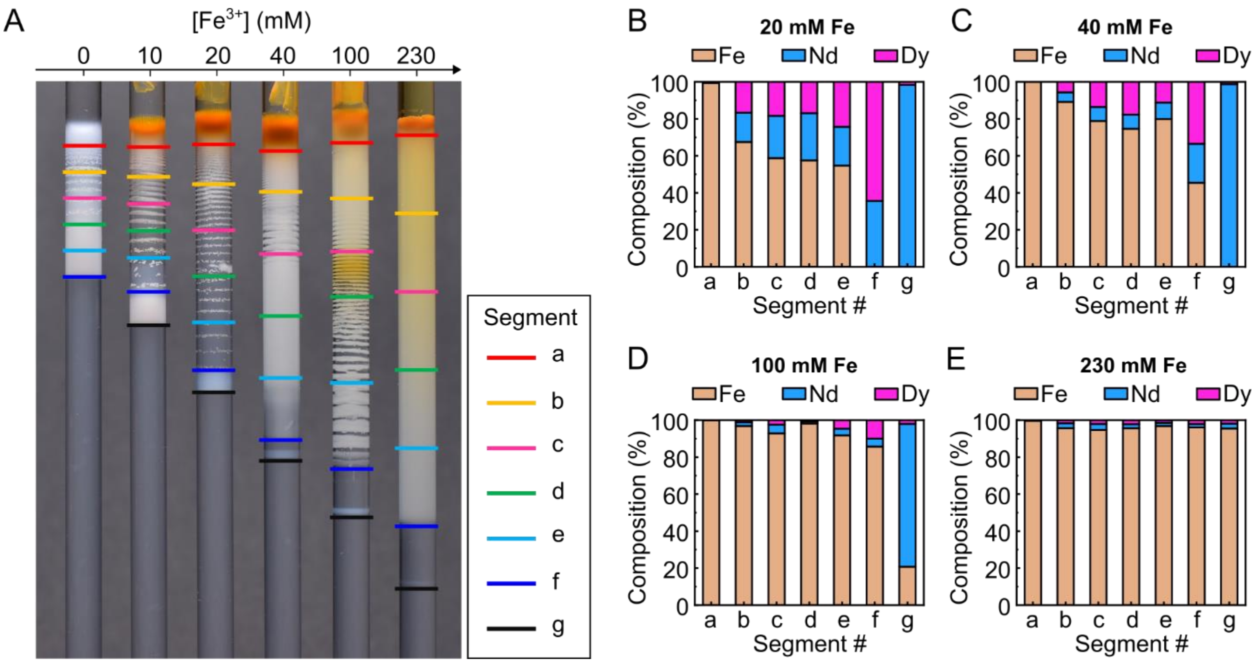

Figure S18. (A) Photograph of resulting precipitation patterns for various Fe concentrations superimposed with colored bars indicating the segmented sample. (B-E) Molar composition along the precipitate from a mixed-salt experiment with a mixed-reactant gel measured by ICP-MS. (For Figure 4G). positions.

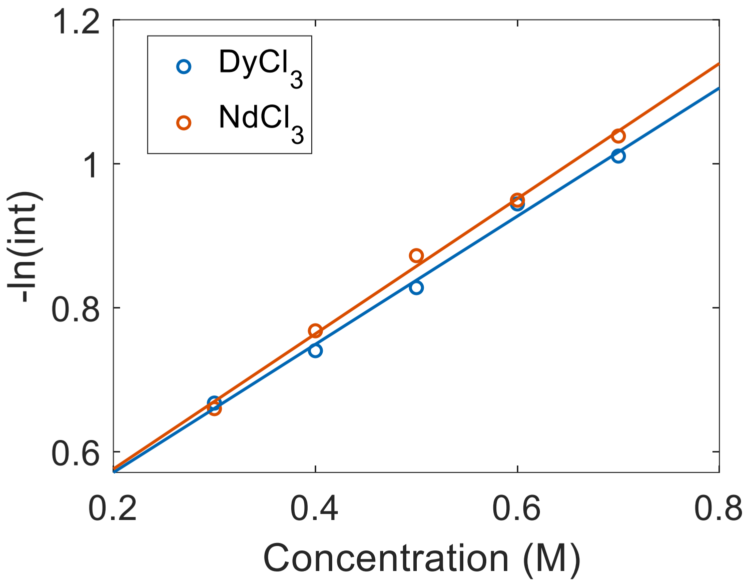

Figure S19. X-ray imaging calibration curves for $NdCl_3$ and $DyCl_3$ solutions.

Table S1. List of parameters used in the reaction-diffusion models. The K_{sp} values of Nd(dbp)$_3$ and Dy(dbp)$_3$ were obtained from literature.[6]

Parameter	Solution domain	Gel domain
D_{Nd} [m^2/s]	1.0×10^{-8}	5.4×10^{-10}
D_{Dy} [m^2/s]	1.0×10^{-8}	5.1×10^{-10}
D_{dbp-} [m^2/s]	6.92×10^{-10}	6.92×10^{-10}
$K_{sp(Nd(dbp)_3)}$ [mol^4/m^{12}]	-	1.3×10^{-14}
$K_{sp(Dy(dbp)_3)}$[mol^4/m^{12}]	-	2.9×10^{-18}
k_{Nd}[m^9/(mol^{3}s)]	-	1.0×10^{-5}
k_{Dy}[m^9/(mol^{3}s)]	-	5.0×10^{-3}

3. Supporting Movies

Movie S1. False-colored time-lapse video showing the dissolution front of the Nd-only experiment. The field of view is 2.4 × 2.4 mm^2.

Movie S2. Time-lapse video showing the precipitate patterns for individual Nd^{3+}, Dy^{3+}, and mixed salts experiments. The gel contained 10 mM dbp^-. The salt solution contained 40 mM Nd^{3+}, 10 mM Dy^{3+}, 10 mM Nd^{3+}, and a mixed salt of 40 mM Nd^{3+} and 10 mM Dy^{3+} from left to right. The video was sped up by a factor of 28800.

Movie S3. Time-lapse video showing the precipitate patterns for various Fe^{3+} concentrations. In these experiments, the salt solution contained 0-230 mM Fe^{3+}, 40 mM Nd^{3+}, and 10 mM Dy^{3+}, and the gel contained 10 mM dbp^- and 30 mM OH^-. The Fe^{3+} concentration was 0, 10, 20, 40, 100, and 230 mM from left to right. The video was sped up by a factor of 28800.

4. Supporting References

[1] *Comsol multiphysics*®, v. 5.5. Stockholm, Sweden, COMSOL AB, **2020**.

[2] S. Miyamoto, K. Shimono, *Chem-Bio Inf. J.* **2022**, *22*, 13-20.

[3] F. Ding, W. Xu, G. L. Graff, J. Zhang, M. L. Sushko, X. Chen, Y. Shao, M. H. Engelhard, Z. Nie, J. Xiao, X. Liu, P. V. Sushko, J. Liu, J.-G. Zhang, *J. Am. Chem. Soc.* **2013**, *135*, 4450-4456.

[4] D. Song, E. J. Bylaska, K. M. Rosso, M. L. Sushko, *J. Phys. Chem. C* **2024**, *128*, 5686-5696.

[5] a) H. Liu, T. Chen, X. Zou, C. Qing, R. L. Frost, *J. Raman Spectrosc.* **2013**, *44*, 1609-1614; b) D. L. A. de Faria, F. N. Lopes, *Vib. Spectrosc.* **2007**, *45*, 117-121.

[6] Y. Tasaki-Handa, Y. Abe, K. Ooi, H. Narita, M. Tanaka, A. Wakisaka, *J. Phys. Chem. B* **2016**, *120*, 12730-12735.
